# InAsSb Photodiode-Based Infrared Radiation Thermometer for the Investigation of Droplet Surface Temperature Dynamics Within an Enclosed Combustion Chamber

**DOI:** 10.3390/s25185780

**Published:** 2025-09-16

**Authors:** Louis Karapateas, Emilios Leonidas, Xiangfei Meng, Yufeng Lai, Yang Zhang, Jon R. Willmott, Matthew J. Hobbs

**Affiliations:** 1 Sensor Systems Group, School of Electrical & Electronic Engineering, The University of Sheffield, Portobello Centre, Pitt Street, Sheffield S1 4ET, UK; lkarapateas2@sheffield.ac.uk (L.K.); eleonidas1@sheffield.ac.uk (E.L.); y.lai@sheffield.ac.uk (Y.L.); j.r.willmott@sheffield.ac.uk (J.R.W.); 2 School of Chemical, Materials and Biological Engineering, The University of Sheffield, Sir Robert Hadfield Building, Mappin Street, Sheffield S1 3JD, UK; 3 School of Mechanical, Aerospace and Civil Engineering, The University of Sheffield, Sir Frederick Mappin Building, Sheffield S1 3JD, UK; omeng1@sheffield.ac.uk (X.M.); yz100@sheffield.ac.uk (Y.Z.)

**Keywords:** combustion, fuel droplet, InAsSb photodiode, infrared radiation thermometer, flame dynamics

## Abstract

This study presents a novel approach to analysing the early stages of the combustion process by measuring the surface temperature of a kerosene droplet from its point of ignition through to its evaporation. An indium arsenide antimonide (InAsSb) photodiode-based infrared radiation thermometer (IRT), operating between 3 μm and 11 μm in wavelength, was designed to enable non-contact, low-temperature sensing with an acquisition time of 500 μs. Integrated with a data acquisition unit (DAQ), the instrument captures the transient combustion stages occurring below the droplet’s boiling point of 300 °C. The instrument was assessed against industry standards and demonstrated a measurement uncertainty of ±2 °C, confirming suitability within the performance bounds of commercial instrumentation. The IRT was deployed to measure the temperature of a kerosene droplet within an enclosed combustion chamber upon ignition, in direct comparison with a contact thermocouple. The instrument demonstrated its capability to measure the droplet’s surface temperature changes throughout its early-stage combustion. Furthermore, the wavelength specificity of the IRT eliminates thermal interference from the subsequent flame, a capability which contact thermocouples lack, thereby enabling measurement of the droplet’s temperature in isolation. This study focuses on single-droplet Jet A kerosene combustion under controlled conditions, using a transferable methodology adaptable to other fuels and environments. It supports the use of IRT for non-contact temperature measurement of fuel droplets and early-stage combustion, aiding fuel characterisation and the development of future fuels such as SAF.

## 1. Introduction

In the process of optimising combustion efficiency, reducing carbon emissions, and enhancing safety within aircraft engines, an accurate understanding of temperature within the combustion process is vital to optimising such parameters. Such insights are therefore crucial for evaluating the respective performance and characteristics of the behaviour of different fuels. Fuel droplets, which are tiny spherical particles of liquid fuel formed during atomisation, play a significant role in combustion [1]. They influence mixing, vaporisation, and burning processes, all of which are conditions that can be simulated within experimental combustion chambers. Conducting fundamental studies on droplet combustion paves the way for developing deeper understandings of atomised combustion of different fuels, a process in which droplet surface temperature is a key parameter within single-droplet combustion studies. According to the American and European air safety regulations, all commercial aircraft must be able to successfully conduct high-altitude reignition in the event of a flame-out [2,3]. In order to meet these regulations, it is necessary to conduct droplet combustion studies under representative conditions. Accurate measurement of droplet surface temperatures in these studies provides valuable insights into heat transfer and the droplet vaporisation process, both of which significantly affect combustion efficiency and stability [4,5,6]. These factors are vital for combustion-related applications such as cooling sprays in gas turbine engines, fuel injection in internal combustion engines, and spray drying processes where controlled combustion conditions are essential [7,8].

There are significant challenges to measuring the temperature of a droplet’s surface, primarily due to the difficulty of establishing reliable contact with such a small, dynamic area. Thermocouples have traditionally been used to measure temperature within combustion processes due to their simplicity and widespread availability. However, even when reliable contact can be established, the measurement process can disrupt the droplet’s combustion behaviour, compromising the accuracy of the readings [9,10]. Furthermore, thermocouples present additional challenges, such as relatively slow response times, typically ranging between 5 and 100 ms [11,12], and limited sensitivity. These limitations ultimately restrict their capability to accurately and precisely measure rapid temperature fluctuations [13,14]. An alternative measurement approach would be to utilise thermal imaging, a non-contact measurement technique that eliminates the need for physical contact, thereby preventing interference with the process [15]. However, measurement of lower target temperatures using thermal imaging presents its own specific challenges; cost-efficient and higher-speed silicon-based options are not suited to such measurements. This would dictate the need for either costly, and typically cooled, photodetector-based MWIR cameras or slower bolometer-based LWIR cameras, neither of which would be desirable for use within this application. Furthermore, thermal imaging is also prone to errors caused by reflections, background radiation, and signal interference between adjacent pixels, stemming from the inherent array nature of imaging cameras, which compromises their suitability for accurate temperature measurement.

Another non-contact temperature measurement technique is that of infrared radiation thermometry (IRT), which has long been used as a practical alternative to thermocouples in applications such as process manufacturing and thermal forming [16,17]. For instance, IRTs are commonly employed during the continuous casting process to monitor molten steel temperatures as it solidifies, preventing defects and maintaining uniformity [18]. Unlike thermal imagers, which capture data across multiple points, IRTs measure temperature at a single point with a single pixel, thereby enabling the minimisation of reflections within their design. They are also used in hot rolling mills to measure the surface temperature of steel sheets and bars, enabling real-time adjustments to optimise processing conditions [19]. IRTs enable high-speed temperature measurements through the utilisation of photon detectors that operate based on the photoelectric effect, which is inherently fast. This allows them to avoid physical interference issues associated with thermocouples and achieve response times in the microsecond range [20,21]. This speed advantage allows IRTs to precisely monitor rapid temperature fluctuations, enabling detailed analysis of transient events, such as those in combustion processes.

The speed advantage of IRTs is particularly attractive for combustion applications such as fuel analysis, engine optimisation, and rocket propulsion safety, with direct relevance to biofuels and synthetic fuels. By eliminating contamination risks and ensuring consistent data over extended periods, IRTs are especially well suited to single-droplet combustion measurements. IRTs that incorporate photon detectors can be tailored for operation across specific infrared wavelength bands by selecting a photodiode with a bandgap corresponding to the application wavelength range. This flexibility, combined with the inherently fast response of photon detectors, enables accurate tracking of rapid, transient thermal events in combustion chambers. Such capabilities are particularly valuable for capturing dynamic processes that are difficult to resolve using conventional diagnostic techniques [22,23,24].

Indium arsenide antimonide (InAsSb) photodiodes are an emerging photodetector technology within the MWIR and LWIR spectral regions. They offer a more practical solution for integration within IRTs due to their ability to operate at room temperature. This eliminates the need for expensive and bulky cooling systems, which are typical of traditional HgCdTe-based detectors. Furthermore, InAsSb-based photodiodes have faster response times compared to thermal detectors such as thermopiles, enabling quicker and more accurate measurements in dynamic or fast-transient applications [25,26]. Their longer wavelength operation and fast response time [27,28] makes them especially useful for measurement of the near-ambient temperatures associated within combustion processes [29,30], even below the theoretical boiling point of a droplet [31,32].

This study presents a non-contact IRT based on an InAsSb photodiode, operating across a broad spectral range of 3–11 μm. The experimental arrangement is specifically designed to measure the surface temperature of a standard Jet A kerosene droplet within the range of 50–300 °C and is experimentally validated through studies of droplet combustion behaviour. Our IRT demonstrates high-speed temperature measurement capabilities with an acquisition time of 500 μs, making it particularly effective for the measurement of dynamic combustion processes that demand fast, non-contact, and low-temperature sensing with high-speed data acquisition. Additionally, the IRT offers a distinct further advantage over thermocouples by accurately measuring the droplet temperature due to its wavelength specificity, whereas thermocouples are more indiscriminate in their measurements, often capturing flame temperature alongside droplet temperature. To the best of our knowledge, this is the first non-contact IRT measurement capable of recording the surface temperature of droplets at near-ambient temperatures. This research contributes to the advancement of fuel analysis instrumentation and measurement techniques, offering a promising approach for the development of future fuels and optimisation of combustion processes.

## 2. Materials and Methods

### 2.1. Instrument Design and Characterisation

**Figure 1:**
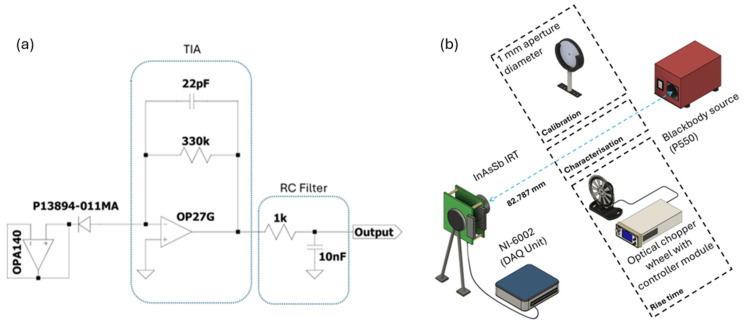
(**a**) Bootstrapped InAsSb photodiode single-stage TIA schematic and (**b**) IRT experimental arrangement for calibration, characterisation, and rise time.

The IRT developed for this study was based around an uncooled InAsSb photodiode (model P13894-011MA) manufactured by Hamamatsu Photonics K.K. (Hamamatsu City, Japan), integrated with a transimpedance amplifier (TIA) circuit, following a configuration similar to that detailed in [33]. The circuit layout shown in Figure 1a illustrates the TIA configuration, in which an operational amplifier is paired with a feedback network comprising a 330 kΩ resistor and a 22 pF capacitor. To reduce high-frequency noise fluctuations, this stage was followed by a first-order low pass filter assembled using a 1 kΩ resistor and a 10 nF capacitor; the overall circuitry’s response time was approximately 383 μs. The photodetector featured a 1.0 mm by 1.0 mm active region, exhibiting sensitivity to infrared wavelengths spanning 3.0 to 11.0 μm, with its peak responsivity occurring at 5.6 μm.

The key performance characteristics of the IRT, including its dynamic range and noise response as a function of target temperature, were evaluated using a blackbody calibration source (Landcal P550, AMETEK LAND, Dronfield, UK). Directly in front of the furnace, a 1 mm aperture was employed to accommodate the submillimetre droplet, as further detailed in Section 2.2. The temperature of the blackbody was incrementally varied from 50 °C up to 300 °C in steps of 50 °C. No optical elements are present between the detector and the blackbody source, ensuring a clear and unobstructed optical path as seen in Figure 1b. The output from the IRT was fed into a National Instruments USB 6002 data acquisition (DAQ) unit (Austin, TX, USA), which recorded the analogue voltage at a sampling rate of 500 μs. Data collection and processing were handled using FlexLogger software (FlexLogger 2024 version Q3), which also enabled assessment of the IRT’s noise behaviour at various integration times, specifically 500 μs, 50 μs, 5 ms, and 50 ms.

The IRT was calibrated radiometrically using Planck’s law, which describes the spectral radiance (*L_b_*) emitted by an ideal blackbody, as shown in Equation (1) [34]. This formulation includes the radiation constants, *c*_1_ and *c*_2_, with integration carried out over the wavelength range defined by the lower and upper bounds, *λ*_1_ and *λ*_2_. The InAsSb photodiode responds to incident radiation through generation if a photocurrent proportional to *L_b_*, which in turn determines the output voltage (*V*). To accurately relate voltage to temperature, a calibration procedure involving five discrete temperature points was conducted, from which a Look-Up Table (LUT) was constructed to map voltage readings to their corresponding thermal values.

(1) $$L_{b}\left(\lambda ,T\right)=\frac{c_{1}}{\lambda^{5}(e^{c_{2}/\lambda T}-1)}$$

Real-world objects are not ideal radiators, thus impacting upon the practical application of Equation (1) when performing temperature measurements using an IRT. The efficiency with which a material’s surface radiates thermal radiation is known as its emissivity and is measured relative to an ideal blackbody at the same temperature; ideal blackbody radiators have an emissivity of 1. All real-world objects therefore have an emissivity of less than 1, meaning that this parameter needs to be accounted for when performing temperature measurements. Within the framework of radiometric analysis, spectral emissivity (*ε*) denotes the ratio of a material’s spectral radiance (*L*) to that of a blackbody reference (*L_b_*) evaluated at a specific wavelength (*λ*) and temperature (*T*). This dimensionless quantity, presented in Equation (2), encapsulates the deviation of real surfaces from ideal blackbody behaviour.

(2) $$\varepsilon \left(\lambda ,T\right)=\frac{L(\lambda ,T)}{L_{b}\left(\lambda ,T\right)}$$

The IRT’s field of view (FOV) was measured to have a target-to-source ratio of 5 to 1. This measurement was obtained by measuring the output of the IRT with a series of apertures of different diameters positioned in front of the blackbody reference furnace. The nominal FOV, defined as the region capturing 98% of source-emitted radiance relative to the paraxial image at the system’s field stop, was determined. Beyond this, size-of-source effect (SSE) analysis verified that extraneous radiation exerted no appreciable influence on measurement accuracy.

The root mean square (RMS) noise of the IRT was quantified by calculating the standard deviation of its temperature-calibrated signal. To benchmark the instrument’s noise performance, the resulting values were compared against the conventional RMS noise threshold of ±0.5 °C, as typically specified for commercial-grade systems [35,36]. Data acquisition was performed using the NI DAQ 6002 module, operating at its default sampling interval of 500 µs over a one-second measurement window. The recorded voltage signals were subsequently converted to temperature values in degrees Celsius, with the standard deviation at each calibration point serving as the RMS noise metric. To assess the influence of temporal averaging on noise suppression, moving average filters were applied to the raw voltage data using integration windows of 500 µs, 1 ms, 5 ms, 20 ms, and 50 ms.

The IRT’s response time was characterised by measuring the rise interval of its output voltage, defined as the duration required for the signal to transition from 10% to 90% of its maximum value. This was achieved using an MC2000B-EC optical chopper (Thorlabs, Newton, NJ, USA), positioned between the fibre optic input and the blackbody source, as shown in Figure 1b. The chopper wheel, rotating at 600 Hz, cyclically interrupted the optical path to the furnace cavity. The resulting modulated signal was recorded via an oscilloscope operating at a temporal resolution of 500 µs.

### 2.2. Droplet Closed Environment Combustion Chamber

**Figure 2:**
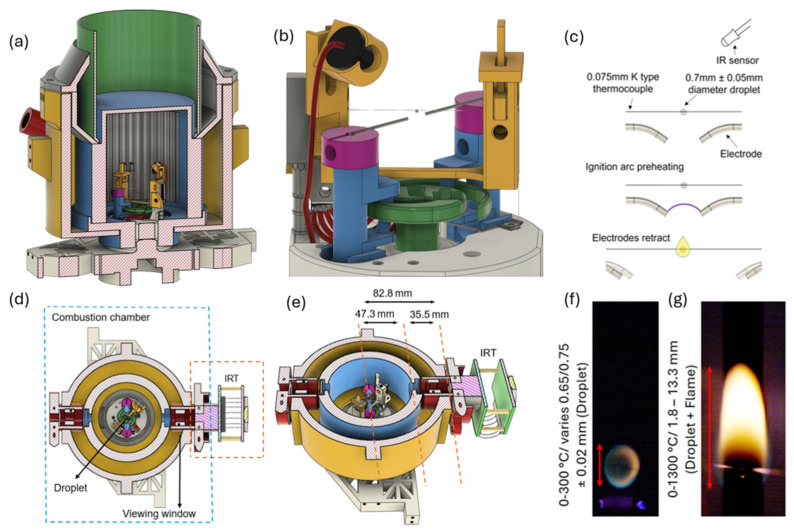
(**a**) Closed combustion chamber/droplet setup, (**b**) ignition system, (**c**) spark ignition sequence, (**d**,**e**) detailed measured IRT positions, and (**f**,**g**) droplet ignition dynamics and diameters following ignition.

The experimental enclosed combustion chamber setup, designed to recreate high-altitude reignition conditions, is shown in Figure 2a,b. Although not used within this work, this setup incorporates a pump and liquid nitrogen input, allowing for independent adjustments of pressure and temperature, which range from 20 kPa to 101 kPa and 253 K to 291 K, by controlling the air valve and the volume of liquid nitrogen in the cooling tray. A flame detector module is integrated into the system to deactivate the ignition sparks as soon as the soot flame appears, ensuring minimal energy input and consistent results. A 0.075 mm diameter K-type thermocouple, connected to a National Instruments (NI) card via a thermocouple transmitter, is used to collect droplet temperature data.

During each experiment, a fuel droplet approximately 0.7 mm ± 0.05 mm in diameter (shown in Figure 2c) is suspended onto the thermocouple using a 10 μL micro syringe pipette. A constant spark, delivering about 20 Js^−1^ of power, is discharged beneath the droplet until the soot flame appears. Throughout the experiments, the positions of both the suspended droplet and the ignition spark are fixed to maintain consistency. Once ignited within the sealed combustion chamber, the combustion process is observed through a 25 mm diameter viewing window integrated into the chamber. Unlike the method used in [2], a quartz window is not included (Figure 2d,e); the inclusion of such a window would block the wavelengths that the InAsSb photodiode is sensitive to. A 3D-printed mount was created to position the IRT within the sealed chamber, not externally, as shown in Figure 2d,e, highlighting the detector distance and optical clarity. Positioned 82.8 mm from the combustion chamber, the IRT was calculated to be sighted upon a target area of approximately 16.5 mm in diameter, based on the IRT’s measured FOV of 5:1. The ignition spark initiates droplet heating, marking the beginning of the combustion process. Heating of the jet fuel droplet subsequently progresses, causing the more volatile components of the jet fuel to vaporise, which forms a vapour-rich envelope around the droplet and establishes a concentration boundary layer. Once enough heat accumulates, a flame envelope develops around the droplet. A Keys Flame Sensor Module, which incorporates a silicon photodiode, is used to detect the presence of the flame. Once the flame is detected, this sensor sends a signal that shuts off the ignition spark, marking the end of the ignition delay and the onset of self-sustained combustion. The consumption of the volatile components causes the less-volatile components to migrate to the droplet’s surface, continuing the gasification process [37,38].

Two representative stages of droplet flame development under ambient, room-temperature conditions are shown in Figure 2f,g. In this sequence, Figure 2f depicts droplet ignition, whilst Figure 2g shows the self-sustained flame, together illustrating typical kerosene flame morphology at different stages of the process. The ignition and combustion sequence were recorded using a Photron FASTCAM SA4 (Photron Ltd., Tokyo, Japan) high-speed camera operating at 2000 fps, synchronised with the ignition system via an Arduino circuit board.

To measure the droplet’s diameter, the size of each pixel was calibrated using the known thickness of the suspension thermocouple wire, and the flame diameter was thereby calculated accordingly [39]. During this period, the droplet’s diameter was expected to vary between 0.65 mm and 0.75 mm due to the puffing phenomenon caused by heat accumulation, with an approximate 0.02 mm increase in diameter being observed [40]. The small size of the droplet relative to the IRT’s FOV was accounted for during instrument calibration by placing a 1 mm diameter target aperture positioned in front of the furnace within the setup shown in Figure 1. The maximum horizontal flame diameter, shown in Figure 2g, ranges from 1.8 mm to 13.3 mm when compared with the droplet’s surface in Figure 2f. The measured surface temperature of the droplet is theoretically expected to remain below the boiling point of the fuel, typically under 300 °C. This aligns with values reported in the literature, where temperatures generally fluctuate around this range [41,42,43,44]. Furthermore, at lower temperatures, below circa 100 °C, the non-linearities inherent to the lower detection limit of the IRT response were accounted for during the calibration process.

Whilst the thermocouple wire enables measurement of the internal droplet temperature, it is limited to a fixed point, as shown in Figure 2b. Moreover, the precise measurement location can vary between different thermocouple wires, potentially introducing inconsistencies and making data comparison unreliable. To address this and to ensure alignment between the two measurement systems, manual synchronisation was performed by matching their timestamps, as the thermocouple’s data acquisition began after the IRT had already started recording.

## 3. Results and Discussion

### 3.1. IRT Characterisation

**Figure 3:**
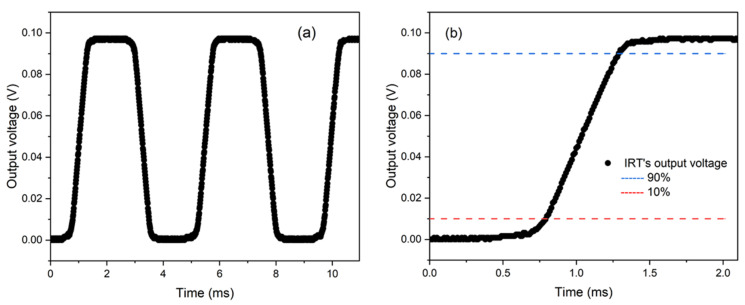
(**a**) Response time analysis and (**b**) focused rise time analysis with a chopper wheel.

To confirm the IRT’s suitability for radiometric measurements at a temporal resolution of 500 µs, its performance was assessed using the experimental procedures detailed in Section 2.1. When evaluating the IRT’s response to a rapid signal variation (Figure 3a) and its corresponding rise time characteristics (Figure 3b), the analogue output voltage rise time was determined to be 350 μs, exceeding the temporal resolution of the digital acquisition system, which operates at 500 µs intervals. This ensures compatibility with the subsequent data acquisition rate of the DAQ unit.

**Figure 4:**
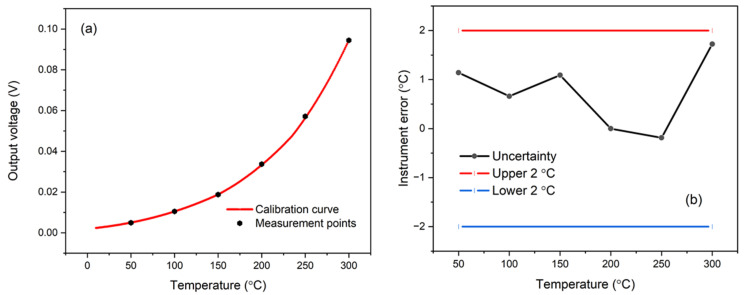
(**a**) Variation of IRT output voltage with target temperature and (**b**) quantified measurement uncertainty across 50–300 °C.

The mean output voltage of the IRT was measured across target temperatures from 50 °C to 300 °C, as shown in Figure 4a. The data demonstrates that both the raw and calibrated voltage responses conform to the spectral behaviour predicted by Planck’s law. Calibration was conducted at discrete temperature points of 51 °C, 102 °C, 149 °C, 198 °C, 251 °C, and 303 °C, with the resulting calibration uncertainty profile shown in Figure 4b. The IRT exhibited a measurement uncertainty of ±0.25% °C + 2 °C, placing its performance within the tolerance range typically specified for commercial-grade instruments.

**Figure 5:**
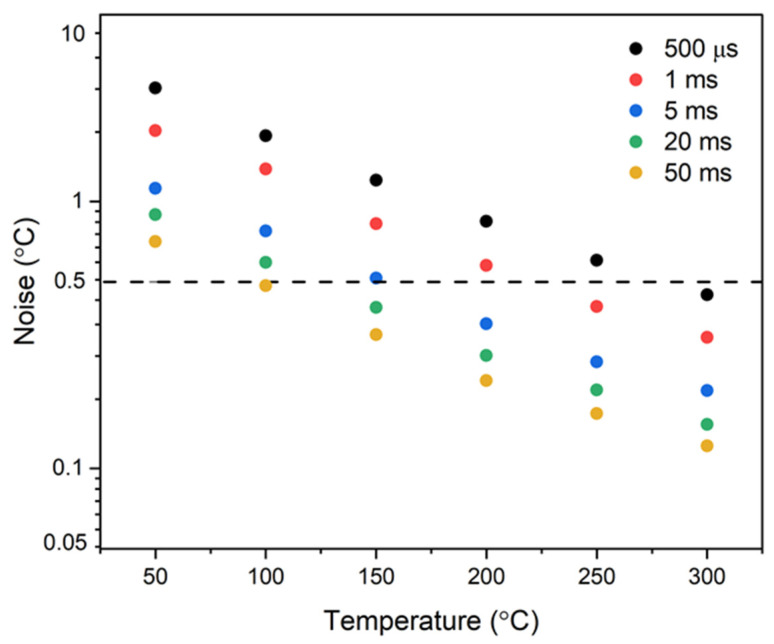
RMS noise variation with target temperature for IRT integration times spanning 500 µs to 50 ms.

The RMS noise profile of the IRT is shown together with a 0.5 °C reference threshold, typical of commercial instruments, indicated by the dotted horizontal line, in Figure 5. At an acquisition interval of 500 µs, this noise criterion was satisfied at a target temperature near 300 °C. Increasing the integration time yields improved noise performance, lowering the temperature required to meet the 0.5 °C threshold to approximately 150 °C at 5 ms and below 100 °C at 50 ms. These findings indicate that the IRT’s noise characteristics are consistent with those of standard commercial thermocouples, affirming the adequacy of its performance at short integration times.

An uncertainty budget was developed to quantify the measurement uncertainty associated with the IRT. This budget incorporates discrete contributions arising from the instrumentation employed during calibration and characterisation procedures alongside those intrinsic to the measurement process. The constituent sources of uncertainty, encompassing both equipment-related and procedural factors, are detailed in Table 1.

At a source temperature of 100 °C, the total measurement uncertainty associated with the InAsSb IRT was quantified as 1.73%. This value predominantly reflects two sources: the intrinsic variability observed in the IRT output, and the interpolation error incurred during the conversion from voltage to temperature. Contributions from the calibration apparatus were assessed to be negligible and may be excluded without compromising the integrity of the uncertainty estimate. Consequently, the uncertainty characterisation for the InAsSb IRT may be streamlined by considering only the aforementioned dominant sources, with the combined uncertainty reported as the root sum square of their respective contributions.

### 3.2. Surface Temperature Measurements of the Droplet

**Figure 6:**
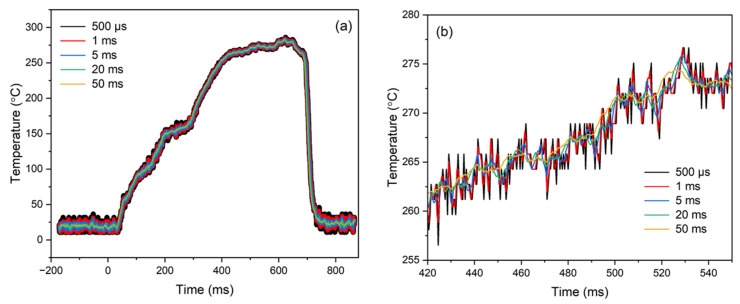
(**a**) Surface temperature measurement of the droplet over integration times between 500 µs and 50 ms and (**b**) subset of droplet surface temperature measurement between 420 ms and 550 ms.

The instrument’s response time and noise performance substantiate its suitability for non-contact, low-temperature measurement applications, making it ideal for measuring the surface temperature during droplet combustion within a sealed combustion chamber. The IRT was subsequently incorporated into the experimental setup (Figure 2), where ignition of a standard aviation Jet A kerosene droplet was initiated; the measured temperature of the droplet under ambient condition is shown at the raw measurement acquisition time of 500 µs in Figure 6a. Overlaid averaged 1–50 ms measurements have been included to illustrate the overall trend in average temperature, capturing various stages throughout the droplet’s combustion.

From −172 ms to 0 ms, the droplet has not yet been ignited; the IRT is therefore only measuring the temperature of the unignited droplet. From 0 ms, which marks the time at which the ignition mechanism is triggered, to 20 ms, the continued measurement of the droplet’s unignited temperature indicates the delay in the heat transfer mechanisms within the droplet. Following this delay, at 20 ms, spark ignition begins, and the preferential gasification of the more volatile components within the fuel, such as n-alkanes and low-molecular-weight iso-alkanes [45], becomes evident from the increase in the droplet’s surface temperature up until 200 ms. Within this period, energy from the spark is diffused into the surroundings and onto the droplet, causing more liquid fuel to evaporate; this results in an associated decrease in the air-to-fuel ratio within the flammable premixed gas zone. Once a sufficient temperature is reached, the premixed gas zone ignites, initiating a self-sustained combustion process driven by the aforementioned more volatile components [46,47]. From 200 ms to 400 ms, the surface temperature reflects a transitional phase in the gasification process, transitioning from dominance by the more volatile components to that of the less volatile ones, such as cycloalkanes, aromatics, and higher-molecular-weight hydrocarbons [48]. The observed reduction in the rate of temperature increase suggests that energy input is increasingly directed towards heating these less volatile components. At 400 ms, the gasification process driven by the less volatile components begins, and the droplet’s surface temperature reaches a steadier value; this can be observed within Figure 6b. After the depletion of the droplet, a sudden decrease in the surface temperature is observed from 650 ms, and the measured temperature subsequently falls back to that of the background temperature; there is no longer any droplet to be measured.

The peak-to-peak variation within the measured data within Figure 6 is relatively small; for the raw 500 µs acquisition time, this equates to an RMS noise of approximately 1.29 °C at circa 265 °C. This noise is slightly larger than that shown within the noise measurement in Figure 6, with this slight increase due to the relatively small size of the droplet. This indicates that there is a relatively low level of temperature fluctuations throughout each of these stages of the droplet’s combustion, suggesting that the combustion process is stable and relatively uniform. These minimal transients support the interpretation that the process is consistent and controlled rather than significantly unstable; this stability is typical of droplet combustion [49].

**Figure 7:**
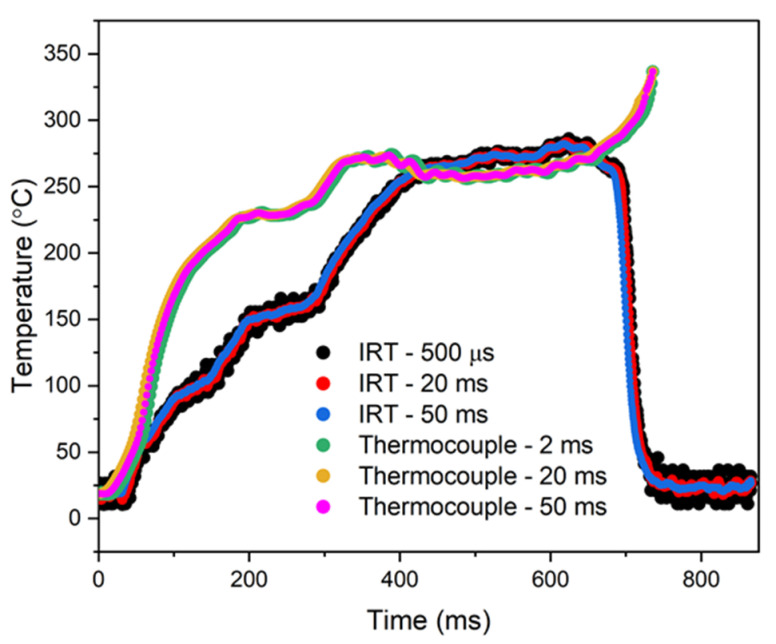
Comparison of the surface temperature measurement of the droplet between the thermocouple and the IRT over the course of the combustion process.

A direct comparison between the thermocouple and IRT measurements is shown in Figure 7, with 20 ms and 50 ms integration time overlays applied to both raw datasets. These overlays were introduced to allow for a fair and comparative analysis between the thermocouple and IRT measurements, thereby reinforcing the consistency of the temperature measurement data. From 0 ms to 20 ms, prior to the onset of ignition at 20 ms, the readings from both the IRT and the thermocouple remain constant and low, corresponding to the measurement of the unignited droplet’s temperature during this pre-ignition phase.

From 20 ms to 50 ms, a sharp rise in temperature can be observed, which coincides with the onset of spark ignition; both the IRT and thermocouple register this increase. Between 50 ms and 400 ms, the measured temperature continues to rise, but discrepancies start to emerge between the two instruments. Whilst both instruments indicate the same trend, the absolute temperature readings differ by up to 100 °C. It is hypothesised that this divergence can be attributed to differences in measurement principles and how they respond to surface emissivity. During this period, the droplet remains in a liquid state and exhibits relatively higher reflectivity. Although liquids can exhibit high intrinsic emissivity under ideal planar conditions, in practice, temperature dependence, surface curvature, dynamic deformation, and specular reflection collectively impact upon the effective emissivity observed by the IRT. It is therefore assumed that the effective emissivity of the droplet is therefore lower at this point of the combustion process. This reduction in radiative output is particularly significant at lower temperatures, where the emitted infrared wavelengths are longer and measurement error due to unknown emissivity becomes more pronounced [50]. We hypothesise that the IRT, which relies upon the measurement of emitted infrared radiation, underestimates the true surface temperature when emissivity is not corrected for. In contrast, thermocouples, being in direct physical contact with the droplet, are unaffected by emissivity, which explains the observed discrepancy. At approximately 400 ms, the droplet reaches approximately 250 °C and puffing behaviour begins (Section 2.2). This phase involves surface roughening and the formation of small particulates as the less volatile components vaporise. These changes disrupt specular reflections and contribute to an increase in effective emissivity. Even under these conditions, flame emissivity remains below unity and can vary spatially depending on soot content and optical thickness [51].

From 400 ms to 650 ms, the temperature measurements from both instruments converge more closely. During this interval, soot begins to form on the droplet surface, altering the optical characteristics of the radiating region. Soot particles are highly absorptive across the infrared spectrum and behave closer to blackbody emitters. The effective emissivity therefore increases substantially with soot development, producing stronger and more uniform infrared emission, but remains slightly below unity, particularly in small-scale flames with moderate optical thickness [52]. This increase in emissivity reduces the IRT measurement error and leads to improved agreement with thermocouple measurements (Figure 7). Whilst the enclosed chamber provides some enhancement of apparent emissivity through internal reflections and radiation exchange, it was not specifically designed to be a blackbody cavity. The system therefore cannot be considered emissivity-independent. The observed convergence in measured temperatures is instead attributed to the increasing effective emissivity as combustion progresses [53].

At 650 ms, a notable difference emerges between the temperature readings of the IRT and the thermocouple; the thermocouple reading increases, whilst the IRT reading decreases. It is during this period that the droplet has reached its burnout phase and has now fully evaporated. The decrease in the IRT reading suggests that there is a lack of detectable signal emitted within the IRT’s sensitive wavelength range of 3–11 µm. In contrast, the thermocouple continues to register thermal activity from the remaining hot gases or particles, as it is unaffected by radiative spectral limitations [54,55]. The sensor outputs for both the IRT and the thermocouple confirm the ability to track the droplet’s surface temperature, which remains below the boiling point of 300 °C. Whilst consistent with previous studies [56,57,58], the differences between the two measurements reflect the spectral specificity of the IRT, enabling distinction between the temperature of the droplet and the subsequent flame. The IRT, therefore, offers the advantage of distinguishing between the droplet and the flame, enabling measurement up to the droplet’s evaporation phase, whereas the thermocouple continues to measure the flame temperature.

These measurements demonstrate the potential of IRTs to characterise the temperature of droplets during combustion, thereby enabling a deeper understanding of what is happening within each time interval throughout the combustion process, particularly during its initial stages. High-speed, non-contact IRTs are therefore well-suited to become valuable tools for measuring droplet surface temperature during combustion. Whilst the continued use of thermocouples will continue to play an important role within fuel characterisation studies, the ability of IRTs to distinguish between droplet and flame temperatures provides additional insights. Their ability to measure droplet temperature without the need for physical contact has distinct advantages over thermocouples; they have no impact upon the droplet’s natural behaviour during the combustion process. High-speed IRTs therefore have the potential to offer greater insights into combustion and fuel-burning processes, which will ultimately advance the field of fuel characterisation and fuel development.

## 4. Conclusions

This study showcases the effectiveness of a high-speed, non-contact IRT for measuring droplet surface temperature during combustion. Operating within a wavelength range of 3–11 μm with an acquisition time of 500 μs, the IRT captures dynamic temperature data without disrupting the droplet’s natural behaviour. A key strength of this approach lies in its ability to measure low temperatures approaching ambient conditions; this is essential for understanding the initial stages of the combustion process. The IRT’s wavelength specificity was shown to offer a distinct advantage over thermocouples, enabling droplet temperature measurements while avoiding interference from flame radiation, an issue thermocouples face due to their inherently indiscriminate approach to temperature measurement. The IRT’s capability to record distinct phases throughout the early stages of the combustion process further validate its suitability for fuel analysis and combustion efficiency studies. By addressing the limitations of traditional methods, this approach offers a practical solution for supporting the development of future sustainable fuels and optimising combustion processes.

## Figures and Tables

**Table 1:** Calibration and measurement uncertainty calculations.

	Target/Reference Temperature (°C)	Blackbody and Thermocouple Contribution to Uncertainty (%)	NI 6002-DAQ-Induced Calibration Uncertainty (%)
Calibration Uncertainties	100	0.3/0.4	0.003
	Target/Reference Temperature (°C)	IRT Measurement Variability (%)	Mean-IRT Interpolation Deviation (%)
Measurement Uncertainties	100	1.73	1.43

## Data Availability

All relevant data are shown in the paper or could be recreated by following the methodology in the paper.
